# Improved Dose Conformity for Adjacent Targets: A Novel Planning Technique for Gamma Knife Stereotactic Radiosurgery

**DOI:** 10.7759/cureus.3057

**Published:** 2018-07-27

**Authors:** Qianyi Xu, Jinyu Xue, Gregory Kubicek, David Mulvihill, Steven Oh, Warren Goldman, Alan Turtz, Leonard Kim

**Affiliations:** 1 Radiation Oncology, MD Anderson Cancer Center at Cooper, Mount Laurel, USA; 2 Radiation Oncology, New York University Langone Medical Center, New York, USA; 3 Radiation Oncology, MD Anderson Cancer Center at Cooper, Camden, USA; 4 Neurosurgery, Cooper Medical School, Camden, USA; 5 Neurosurgery, Cooper Medica, Camden, USA

**Keywords:** gamma knife, stereotactic radiosurgery (srs), gammaplan, inverse planning

## Abstract

Purpose

In the current Gamma Knife (GK) planning system (GammaPlan, version 10.2, Elekta AB, Stockholm, Sweden), multiple adjacent brain metastasis (BMs) had to be planned sequentially if BMs were drawn separately, leading to less conformal target dose in the composite plan due to inter-target dose contribution and fine-tuning of the shots being quite tedious. We proposed a method to improve target dose conformality and planning efficiency for such cases.

Methods and Materials

Fifteen patients with multiple BMs treated on the Leksell GK Perfexion system were retrospectively replanned in the Institutional Review Board (IRB) approved study. The recruitment criterion was all the BMs should be entirely encompassed within the maximum dose grid allowed in the GammaPlan. The BMs were first planned sequentially as routine clinic cases. The contours of the BMs were then exported to the VelocityAI (Varian, CA, USA) to generate a composite contour after a union operation, and all the BMs were planned again simultaneously using this composite contour in the GammaPlan. The inverse planning (IP) was employed in both methods with the same treatment time allowed for a fair plan comparison. Dose evaluation was performed in the VelocityAI with all planning magnetic resonance (MR) images, structure set and dose were exported to the VelocityAI. The dosimetery parameters, including conformality index (CI), V20Gy, V16Gy, V12Gy, and V5Gy, were compared between the two methods.

Results

The planning results from both methods were reviewed qualitatively and quantitatively. The proposed method exhibited superior CI, except for an outlier case with very tiny BMs. The mean and standard deviation (std.) of the Paddick CI for all patients were 0.76±0.11 for the proposed method, comparing to 0.69±0.13 for the sequential method. The V20Gy, V16Gy, V12Gy, and V5Gy for the proposed method were 10.9±0.9%, 9.5±10.2%, 6.2±16.4% and 3.3±21.8%, all lower than those from the sequential method.

Conclusions

The proposed method showed improved target dose conformality for all cases except for very tiny BMs. Planning efficiency is considerably better with the combined target technique. The improved dose conformality will be beneficial to patients in long term with lowered risk of radiation necrosis after GK stereotactic radiosurgery (SRS).

## Introduction

It has been reported that brain metastasis (BMs) develop in about 10%-30% of cancer patients [1]. Current management of BMs includes surgery, radiotherapy, chemotherapy, or combination of these modalities. In radiotherapy, traditional treatment of BMs used to be whole brain radiation therapy (WBRT) and/or stereotactic radiosurgery (SRS). More and more patients with BMs have been treated with SRS for the favored survival rate and quality of life (QOL) [2]. Gamma Knife (GK) has been the gold standard of single fraction SRS with superior accuracy and the proven effectiveness and efficiency.

GK sub-millimeter accuracy is achieved through immobilizing the patient head on the couch using a stereotactic frame (G-frame) or a facial mask with optical tracking, depending on the model of machine. Multiple narrow Cobalt-60 beams are converged to treatment isocenters, each of which corresponds to a shot in a GK plan. Forward planning (FP) technique has been widely adopted in GammaPlan that planners manually place shots on the target to paint a desired dose distribution. More than one shot is typically needed to achieve conformal target dose except for those very tiny BMs. As each shot contributes a portion of the target dose, the total target dose is determined by the number, location, size, and shape of the shots. The quality of GK plans highly depends on the experience of planners, as well as the time allowed for planning and treatment.

Inverse planning (IP) option is made available in version 10.0 of GammaPlan to resolve the issue of tedious FP planning [3]. After initial shots are generated automatically by the IP algorithm or manually by planners, the GammaPlan iteratively runs optimization to achieve conformal target dose based on the planning settings. The iteration can be interrupted anytime, if the planning parameters need to be tuned. In the current version of GammaPlan, all BMs need to be planned in sequence with the IP technique, i.e., one target a time. The issue arises for some BMs that are located in close proximity. As significant dose contribution from nearby BMs cannot be taken into account during the IP planning of each individual target; the conformality of each individual BM becomes worse when reviewed in all target/composite mode. This composite dose would be the delivered radiation dose to the patient. Manual adjustment of the shots can be performed to improve the dose conformality by toggling between all targets/single target modes. However, fine-tuning of a number of shots is tedious and time consuming, as more shots are generally created in IP plans in comparison with FP plans [3].

In this study, we proposed a novel method to optimize all the adjacent BMs simultaneously such that the inter-target dose contribution can be taken into account during the IP optimization process. The objective is to minimize the dose overlapping in between targets while achieving good coverage for each individual target. The dosimetry parameters from the proposed method were compared to those from the sequential mode, including the Paddick CI [4], V20Gy, V16Gy, V12Gy, and V5Gy. The proposed technique has shown much better conformality of the target dose as well as planning efficiency.

## Materials and methods

Patient selection

Fifteen patients with BMs treated on the Leksell GK Perfexion system were retrospectively enrolled in the study after the institutional IRB approval. The recruitment criterion was the BMs of the patient entirely encompassed within the maximum dose grid, which was 7.75 cm (maximum voxel size 2.5mm×31 voxels) in our GammaPlan. The dimension (the maximum size in one direction) of the BM varied from 3.0 mm to 43.1 mm (mean and std. of 19.5±10.8 mm, respectively) and the volume ranged from 0.008 cc to 28.9 cc (mean and std. of 5.5±8.1 cc). We chose patients with combinations of the BMs in different sizes and distances between the BMs, and expected our findings would ensure a more general applicability. The administered dose varied from 15 Gy to 20 Gy (mean and std. of 18.3±1.9 Gy, respectively), depending on the size of the tumors.

Planning process in the sequential mode

The IP process for a single BM was well described by Schlesinger et al. It started from the automatic filling of the initial shots by the GammaPlan and the number of the initial shots depended on the setting of the collimator size. The two main planning settings (Coverage and Selectivity) needed to be balanced during the IP process, as they conflicted with each other. We typically started planning with optimization settings of Coverage (0.75), Selectivity (0.25) and Beam-on (0.1). A plan with clinically acceptable coverage and selectivity could be achieved by tuning these parameters. If treatment time was of concerns, Beam-on setting could be increased to reduce the treatment time. The plan was reviewed by the planner after overlaying the BM with the prescription isodose line. If needed, extra shots were manually added after the IP was done to maximize the target coverage without significant dropping of the selectivity. The same planning process was repeated for the rest of the BMs.

Planning process in the combined target mode

As the current version of the GammaPlan does not provide an operation to combine the separate targets, the structure set of the BMs was exported to the VelocityAI to create the planning target. A composite contour set including all the BMs was generated through a union operation and then sent back to the GammaPlan system. A dose grid was chosen to encompass all the BMs. The similar setting of the collimator size was chosen when filling in the initial shots. In some cases, manually adding some extra shots was needed. For example, if a BM was much smaller than the adjacent BM, it might not receive enough initial shots after automatic filling. As mentioned earlier, the quality of the IP plan was highly correlated to the treatment time. For a fair comparison of plans, the same total treatment time as in the sequential mode was allowed for the combined target mode. The IP process was similar to the single target planning, including manually adding a few shots to increase coverage after the optimization was completed.

Dose evaluation

The MR, structure set and dose from both planning modes were exported to the VelocityAI for dose evaluation. For GK plans included a small BM (< 1 cm), the dose was recalculated in the GammaPlan with 0.5 mm resolution before exporting, otherwise, 1.0 mm resolution was used. The prescription for all the targets was revised to 20 Gy in a single fraction for the purpose of comparisons. The V20Gy, V16Gy, V12Gy, and V5Gy were evaluated for both high and low dose spillages. The Paddick CI is defined as:

$CI = TVPIV^2/(TV \times PIV)$

where TV_PIV_ was the volume of the targets receiving prescription dose, TV was the combined target volumes and PIV was the prescription isodose volume.

## Results

**Figure 1:**
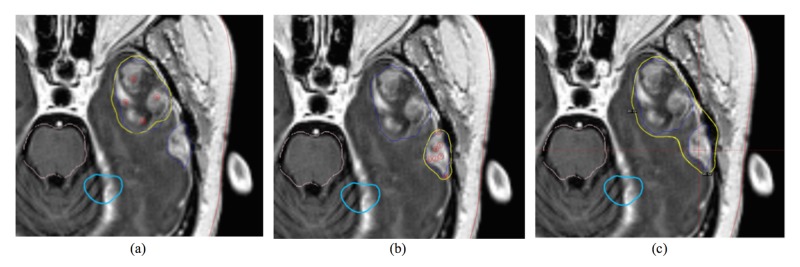
The planning results when brain metastasis (BMs) were planned sequentially In (a) and (b), each BM was planned in the single target mode separately. When reviewed in the all target/composite mode (c), the prescription isodose line between the two BMs spilled over to the normal tissue in between due to inter-target dose contribution and the conformality index (CI) becomes much worse than those appeared in the single target mode.

**Figure 2:**
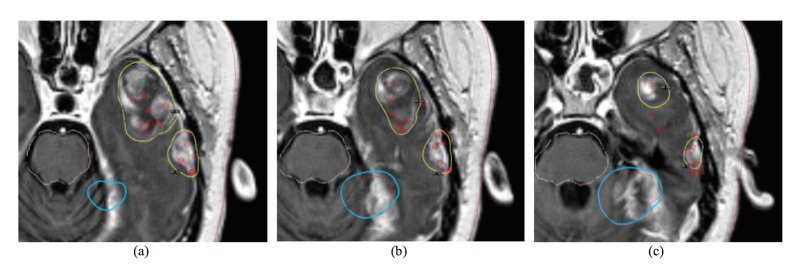
The planning results of the case in Figure 1 using the proposed method In the axial slices from (a) to (c), the prescription isodose line was well separated between the brain metastasis (BMs), and conformality index (CI) was improved from 0.76 to 0.84.

**Figure 3:**
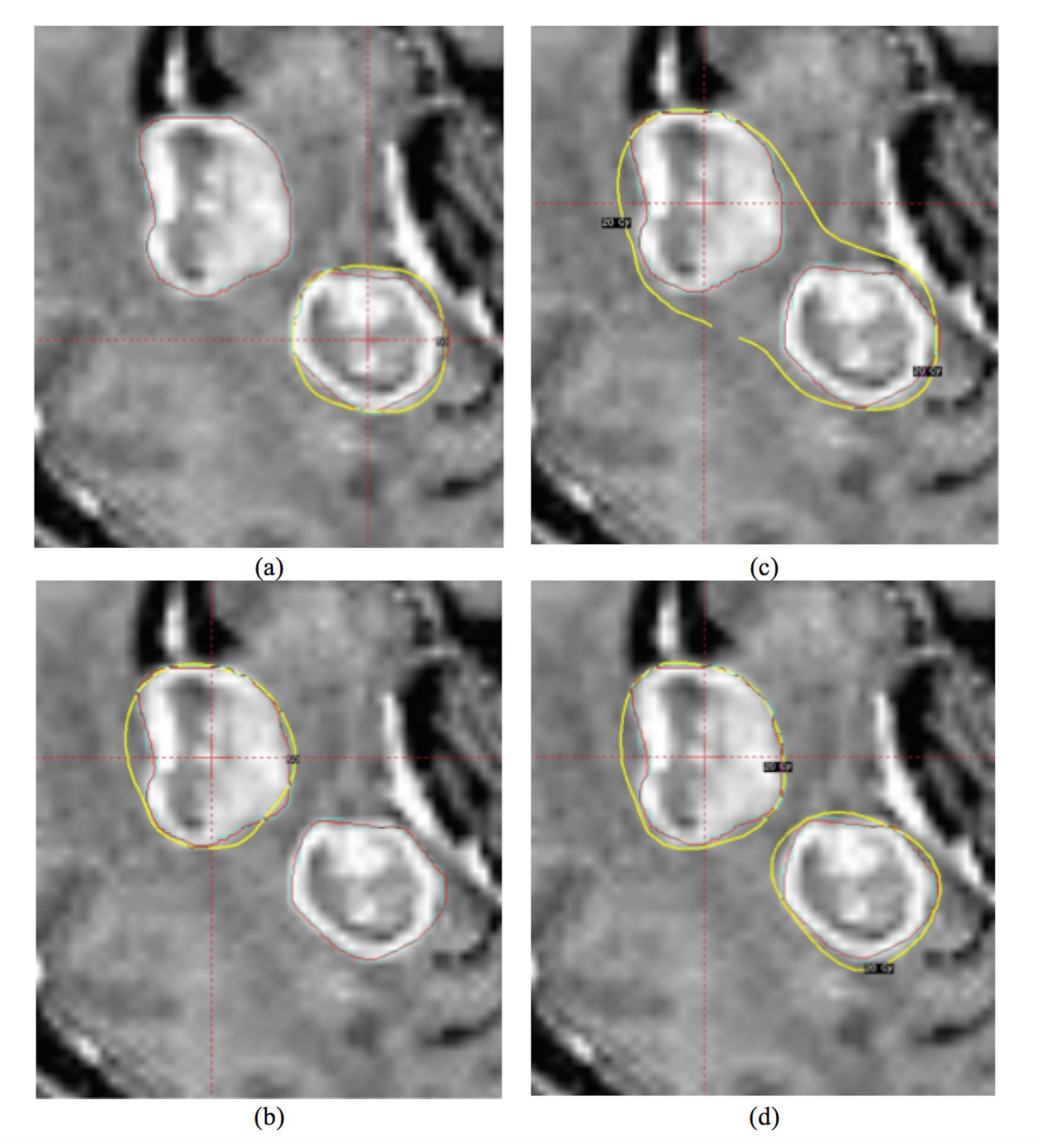
Another example with improved conformality index (CI) (0.6 v.s. 0.79) in the combined target mode The brain metastasis (BMs) were planned in the sequential mode (a and b) and reviewed in the all target/composite mode (c). The two BMs were planned in the combined target mode (d).

The planning results from both sequential and combined target modes were compared qualitatively and quantitatively. Two representative examples are shown in Figures 1-3. The dosimetry parameters from two methods are listed in Table 1. More conformal target dose was observed in the plans from the combined target mode, except for an outlier case. The mean and std. of the Paddick CI for all patients were improved from 0.69 ± 0.13 (sequential mode) to 0.76 ± 0.11 (combined target mode). In the outlier case, it was difficult to distinguish the Paddick CI between the two modes due to the extremely small size of the BMs (0.008 cc and 0.026 cc).

**Table 1:** Comparison of dosimetry parameters between the two methods The parameters were presented as mean ± std.

	Paddick CI	Coverage	V20Gy(cc)	V16Gy(cc)	V12Gy(cc)	V5Gy(cc)
New method	0.76±0.11	98.7±0.69%	13.0±15.3	20.0±22.0	32.5±34.0	129.4±139.7
Sequential mode	0.69±0.12	99.1±0.59%	14.3±16.6	21.4±23.4	34.1±35.5	131.2±139.4

The V20Gy, V16Gy, V12Gy, and V5Gy for the combined target mode were 10.9 ± 0.9%, 9.5 ± 10.2%, 6.2 ± 16.4% and 3.3 ± 21.8%, lower than those for the sequential mode. The improvement was less significant for lower dose, as neither methods aimed to minimize the low dose spillage (e.g., 5 Gy). The target coverage for the sequential mode was slightly higher (99.1 ± 0.59% v.s. 98.7 ± 0.69%). The mean and std. of the treatment time for both methods were very close (97.28 ± 49.36 min for the combined target mode and 100.14 ± 51.60 min for the sequential mode). No significant difference was observed for the selectivity between the two methods (0.74±0.19 for the combined target mode and 0.75±0.15 for the sequential mode).

**Figure 4:**
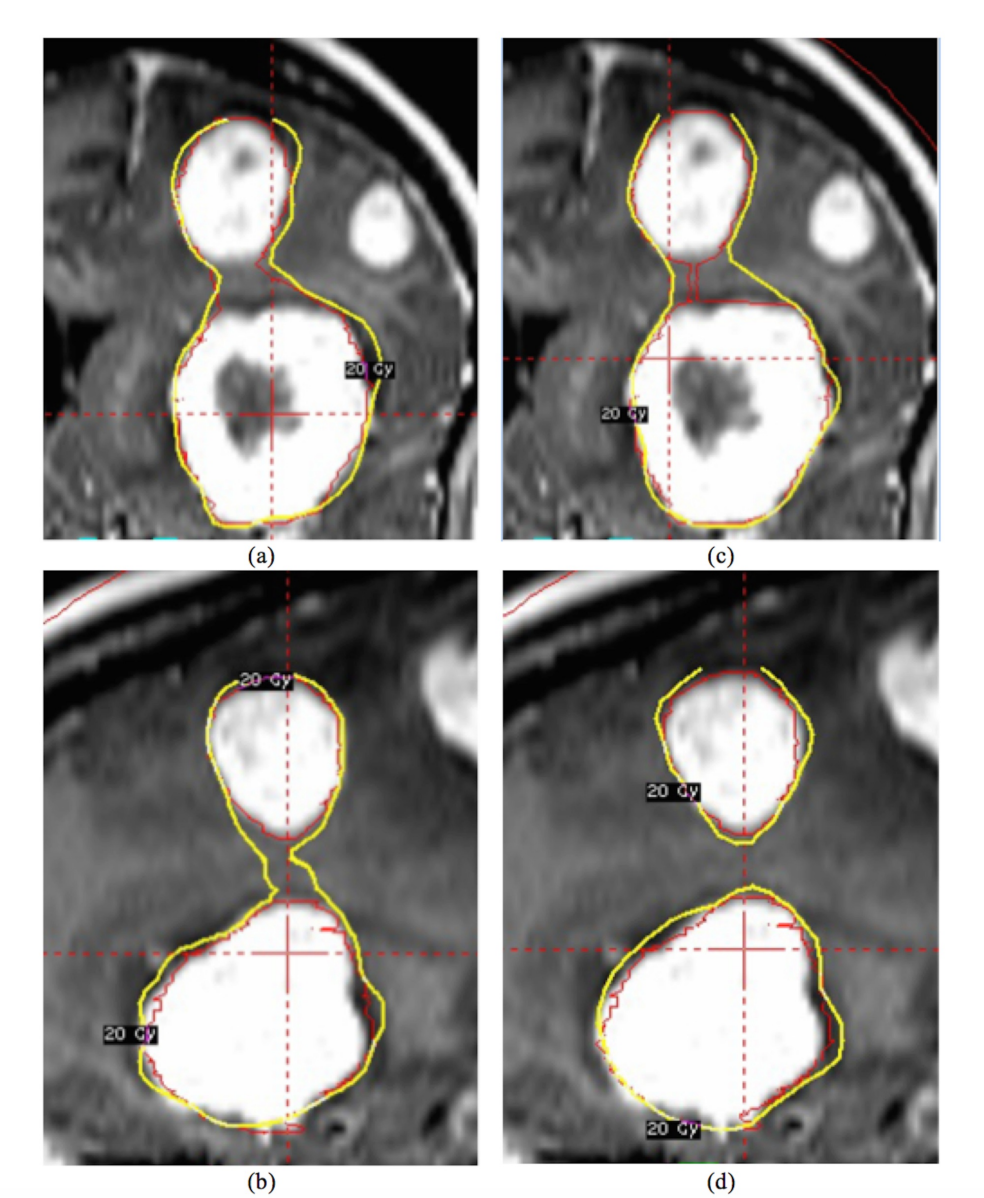
The two brain metastasis (BMs) were adjacent in the superior-inferior (SI) direction and planned in the sequential mode (a) in coronal view and (b) in sagittal view and the combined target mode (c) and (d). A thin inlet was visible in (c) to connect the two brain metastasis (BMs). Although no significant improvement was shown in the coronal view (a and c), a separated prescription isodose line was observed in sagittal view for the combined target mode (d).

Among all the patients except one, at least two BMs were visible in at least one axial slice. For the outlier case, the two BMs were adjacent in the superior-inferior (SI) direction and separated by a few axial slices (Figure 4A). The combined contour set was interpreted correctly in the VelocityAI. After sending back to the GammaPlan, however, the interpolated axial contours were automatically inserted to connect the two BMs (pointed by the arrow in Figure 4A). As the interpolated contours did not represent any treating target, we proposed to manually draw a thin inlet to connect the two BMs to minimize its effect during planning (Figure 4C). The same planning strategies as described above were applied. The plan from the combined target mode had slightly higher coverage (99.2% v.s. 99.0%), as well as improved V20Gy (38.3 cc v.s. 39.8 cc), V16Gy (56.5 cc v.s. 57.8 cc) and V12Gy (88.6 cc v.s. 91 cc), except V5Gy (390.8 cc v.s. 385.4 cc).

## Discussion

Multiple groups have proposed IP techniques for GK treatment planning. Ferris et al. proposed to solve the GK IP optimization problem through nonlinear programming technique [5]. Leichtman et al. reported automated GK planning using polygon clipping and adaptive simulated annealing [6]. Lee et al. used the Nelder-Mead simplex method to find the solution for GK IP opmization [7]. Zhang et al. applied guided evolutionary simulated annealing optimization algorithm to find the optimal shots and further incorporated a plug quality score during optimization to reduce the dose to the critical structures [8]. Wu et al. developed a two-step scheme to determine an optimal number of the shots and further fine-tune the weights using a linear-programming technique [9]. Luan et al. proposed a dynamic GK radiosurgery by combining a traveling salesman problem and constrained least-square optimizations [10]. Despite these pioneering efforts, the IP was not incorporated into the GammaPlan to facilitate the planning process until recent years.

In the current GammaPlan, there were two factors related to the inter-target dose interaction between the BMs. The first factor was obvious that any single BM received contributing dose from the shots of the other BMs, and, in consequence, the dose coverage for the single BM increased in the all target mode. The second factor was tricky as it is related to dose re-normalization (the different maximum point dose) between the single and all target modes. Each BM had a pre-defined maximum point dose in the single target mode before planning started based on the prescription dose and request isodose line. For e.g., the pre-defined maximum point dose was 40 Gy, if the prescription was 20 Gy at 50% isodose line. This was strictly met after the IP process was finished in the single target mode. When being switched to the all target mode, the contributing dose from all other BMs was added to the pre-defined maximum point dose (40 Gy), resulting in a global maximum point dose (>40 Gy). When the TPS forced the global maximum point dose to 40 Gy, the pre-defined maximum point dose for each BM in the single target mode had to decrease accordingly, which led to the reduced coverage of the BM in this mode.

The final coverage of each BM in the all target mode depends on the combination of these two factors. For most cases, the first factor dominated the second one, which led to the increased coverage for the BMs in the all target mode. However, we observed cases that the coverage of a BM decreased when switched to the all target mode, as a significant portion of the global maximum point dose for this BM was contributed from the other BMs. The situation became more complex if there were more than two adjacent BMs. Our method was designed to reduce the complexity by incorporating the inter-target dose contribution into the IP optimization, and hence, a more conformal plan was warranted.

Another key requirement for the GK IP was the planning speed, as planning started after the head frame was placed on the patient's head. Due to the described effect, a manual adjustment of the shots was challenging and time-consuming after the BMs were planned in the sequential mode. The proposed method could reduce planning time significantly in this aspect. However, a third-party software was needed for transferring and processing contours, which took a few minutes using VelocityAI. Also, as multiple BMs were planned simultaneously, different levels of dosing were not feasible in the proposed method. All the BMs in this study were adjacent in the axial plane, except the one in the SI direction. A connecting thin inlet was drawn to avoid the automatically interpolated contours. Although an improved plan quality was observed for the outlier case, more studies are needed to ensure a more general applicability, and eventually incorporation of this new technique in the GammaPlan system.

## Conclusions

The proposed method exhibits improved plan conformality for most adjacent BMs, as well as greater planning efficiency, with the price paid for an extra contour operation. With less irradiated normal brain tissue, the proposed method will be beneficial to patients in the long term with reduced chances of radiation necrosis after GK SRS.

## References

[1] Stewart B, Wild CP (2017). World cancer report 2014. Health (NY).

[2] Kubicek GJ, Turtz A, Xue J (2016). Stereotactic radiosurgery for poor performance status patients. Int J Radiat Oncol Biol Phys.

[3] Schlesinger DJ, Sayer FT, Yen CP, Sheehan JP (2010). Leksell GammaPlan version 10.0 preview: performance of the new inverse treatment planning algorithm applied to Gamma Knife surgery for pituitary adenoma. J Neurosurg.

[4] Paddick I, Lippitz B (2006). A simple dose gradient measurement tool to complement the conformity index. J Neurosurg.

[5] Ferris MC, Lim J, Shepard DM (2003). Radiosurgery treatment planning via nonlinear programming. Ann Oper Res.

[6] Leichtman GS, Aita AL, Goldman HW (2000). Automated gamma knife dose planning using polygon clipping and adaptive simulated annealing. Med Phys.

[7] Lee KJ, Barber DC, Walton L (2006). Automated gamma knife radiosurgery treatment planning with image registration, data‐mining, and Nelder‐Mead simplex optimization. Med Phys.

[8] Zhang P, Wu J, Dean D (2003). Plug pattern optimization for gamma knife radiosurgery treatment planning. Int J Radiat Oncol Biol Phys.

[9] Wu QJ, Chankong V, Jitprapaikulsarn S (2003). Real‐time inverse planning for Gamma Knife radiosurgery. Med Phys.

[10] Luan S, Swanson N, Chen Z, Ma L (2009). Dynamic gamma knife radiosurgery. Phys Med Biol.

